# What is the harm in harmful conception? On threshold harms in non-identity cases

**DOI:** 10.1007/s11017-014-9303-7

**Published:** 2014-09-02

**Authors:** Nicola J. Williams, John Harris

**Affiliations:** 1 Centre for Social Ethics and Policy, School of Law, University of Manchester, Oxford Road, Manchester, Lancashire M13 9PL UK; 2 Faculty of Life Sciences, Institute for Science, Ethics and Innovation, University of Manchester, Oxford Road, Manchester, Lancashire M13 9PL UK

**Keywords:** Threshold harms, Derek Parfit, The non-identity problem, Comparative harms, Non-comparative harms, Reproductive decision making

## Abstract

Has the time come to put to bed the concept of a harm threshold when discussing the ethics of reproductive decision making and the legal limits that should be placed upon it? In this commentary, we defend the claim that there exist good moral reasons, despite the conclusions of the non-identity problem, based on the interests of those we might create, to refrain from bringing to birth individuals whose lives are often described in the philosophical literature as ‘less than worth living’.

## Introduction

Has the time come to put to bed the concept of a harm threshold when discussing the ethics of reproductive decision making and the legal limits that should be placed upon it? This is the question asked by Anna Smajdor in her article in this issue. For her, the answer is a resounding yes. She claims—after outlining the concept of the harm threshold in reproductive decision making, noting its many proponents in the philosophical community, and observing its inclusion in legal and policy documents related to reproduction—that no entity can be harmed by being brought into existence, regardless of the extent to which they will suffer once born.

Smajdor comes to this conclusion by appealing to a well-known literature that supports the notion that the act of creation cannot be identical with the acts of harming and benefiting. For if, in accordance with this literature, she suggests, existence should not be considered to be a ‘real’ predicate—that is, if existence cannot be considered an attribute of an object and thus fails to add to the concept of a thing—no entity can be harmed or benefited by being born. In short, her argument is based on the following simple claim: there does not exist a ‘logical connection between the assertion that some lives are not worth living, and the claim that such people are harmed by being conceived’ [1]. Thus, her article, although she does not choose to characterize it in this way, actually constitutes the suggestion that those who subscribe to a harm based and person affecting account of morality (or of the limits of law) must acknowledge that, in holding this view, they are also required^1^ to accept the conclusions of a remarkably *strong version* of a problem in philosophy, known as the ‘non-identity problem’.

In this commentary, we defend the claim that despite the conclusions of the non-identity problem, there are good moral reasons, based upon consideration of the suffering that would be experienced by the individual concerned, not to decide to bring him or her to birth. We have in mind cases in which a foetus or embryo is likely to become an individual whose life is variously described in the philosophical literature as ‘empty of all the things that make life worth living’ [2], ‘dominated by pain and suffering’ [3], ‘intractably miserable’ [4], ‘not worth living’ [5], or ‘worse than no life at all’ [6].

Our arguments are, however, more than mere commentary. We aim to settle some tenacious misunderstandings of the logic of this corner of moral discourse by exploring and explaining the difference between the use of comparative and non-comparative accounts of harm in non-identity cases and the problems that occur when such accounts are conflated. We thus begin our commentary by providing a reconstruction of the major components of the arguments contained within Smajdor’s article. We then question whether Smajdor’s use of Derek Parfit’s arguments in her own is a charitable one that truly captures the spirit in which they were made. After this, we note that although a threshold account of prenatal harm may not be compatible with comparative accounts of harm, the logical inconsistencies Smajdor associates with this account do not occur on non-comparative accounts of harm, such as the one championed by one of us, John Harris, in his book *Wonderwoman and Superman*.

## Doing away with the harm threshold: reconstructing, summarising, explaining, and situating Smajdor’s position in relation to our own

Smajdor begins her attack on the harm threshold in reproductive decision making by noting the benefits that are often associated with harm-based, person-affecting accounts of morality and the proper limits of legislation. She notes, for example, that although she does not subscribe to the belief ‘that harm to offspring is the sole focus of moral concern in reproductive decisions’ [1], there is something to be said for a harm-based approach to legislation and morality. This can be found, she suggests, in the fact that a focus on the harms our choices may impose on others are more identifiable and less subjective than a focus on deontological principles or impersonal and free-floating harms. She thus suggests that because of this more empirical focus, harm based approaches ‘may be a useful interface between morality and legislation’ [1]. She then observes, however—in recognising the conclusions of the non-identity problem, as have many before her, including ourselves (see, for example, [7, 8])—that this approach seems to offer very few of the benefits it provides in cases of harms to extant persons in the context of reproduction.

This is so because if we limit moral and legal criticism only to those acts which can be said to negatively affect the interests of some particular entity, it turns out that our reproductive decisions will, in many cases, have little or no moral content, despite our intuitions to the contrary. That is, if we also accept the relatively uncontroversial proposition that our coming into existence is highly precarious—‘dependent on the conditions under which we and our descendants procreate, with the slightest difference in the conditions of conception sufficient, in a particular case, to [ensure] the creation of a different future person’ [9].

There are a number of versions of what might be called ‘the precariousness proposition’, which produce slight differences in the kinds of circumstances in which non-identity cases are generated. This is so as the particular version to which one subscribes depends greatly on one’s views concerning what it is that makes one numerical person the same numerical person over time and change. However, the version to which Smajdor appeals in her article seems to be based on an acceptance of the precariousness proposition as it is formulated in the work of Parfit in his two versions of the ‘time dependence claim’ (TDC). As she does not make it clear to which version she subscribes—and this actually makes little difference in the cases she considers—we will assume her allegiance is to the slightly weaker form, which, as Parfit notes, is *in fact true*, although not necessarily so:

TDC 2: ‘If any particular person had not been conceived within a month of the time when he was in fact conceived, he would in fact never have existed’. [10, p. 352]

This version of the precariousness proposition may be interpreted in three different ways. It can be seen, firstly, as a causal claim regarding the importance of our genome for the development of personal identity (understood as psychological connectedness and continuity) by noting, for example, that ‘differences in [genetic] material make for later differences in virtually all aspects of a person: change the sperm, and there will be substantial changes (of both a physical and psychological kind) in the later human being’ [11]. Secondly, it can be viewed as a weak version of Kripke’s origins claim regarding the importance of our material origins for numerical identity on biological accounts of personal identity over time. This interpretation suggests that just as all material things must have their origin in at least some of the matter from which they are constituted, so too must all numerical persons have their origins in, *inter alia*, the genetic material from which they are constituted in order to be considered the same numerical entity [8]. Thirdly, however, one might view it as a temporal or environmental claim regarding the importance of possible epigenetic factors and others flowing from the time, place, and manner of our conception. Time, place, and manner may thus embrace factors such as differences in the method of conception, gestational environment, maternal nutrition, the general external environment, and exposure to potentially teratogenic substances during pregnancy.

Depending on one’s interpretation, it can therefore be noted that non-identity is not simply related to conception and factors tied to the timing of conception, nor, as the Parfit of *Reasons and Persons* perhaps believed, simply to genetic identity [8, 11, 12]. However, Smajdor’s interpretation of the claim does seem to fall into either the causal or Kripkean camp, as she notes numerous times in her article, the importance for personal identity of our being conceived from the particular egg or sperm from which we were conceived. This means that, whilst non-identity may not occur in cases of straightforward prenatal harm, such as when a pregnant woman ingests a teratogen or a foetus is injured in some other way during pregnancy, it will be commonplace in cases of genetic decisions regarding, for example, with whom we choose to procreate and when we wish to do so. For this understanding of the TDC2, when paired with a person affecting account of morality that rests on a comparative account of harm, yields a particular prescription. This is that many reproductive decisions turn out to affect not the interests of persons created as a result of such decisions but their identities, and thus cause no harm to those created as a result.

Indeed, the trap of non-identity is not only evidenced in cases of seemingly harmful conceptions. Many people at the time of World War II, for example, including the parents of one of the present authors, decided to await the result of the war and the defeat of Nazism before conceiving or conceiving again. Such people believed they were somehow acting in the interests of the child that would be born to them, despite the fact that they seemed to be ‘guilty’ of falling into the trap of non-identity. It should be noted too that non-identity poses an ‘intriguing theoretical obstacle’ [13] to questions of intergenerational justice and, as has been most recently noted, to cases of affirmative action and of apology or reparation for historic injustices. For, ironically, were it not for the wrong that was done to the ancestors of those now seeking apology or compensation, the latter would almost certainly never have existed [14].

Smajdor illustrates this point in the reproductive case by providing an example of a woman who is receiving treatment for syphilis and must make a choice between conceiving now and giving birth to a child with congenital syphilis, or waiting until after she has been cured and giving birth to a ‘healthy’ child (free from congenital syphilis). Like Parfit, Smajdor suggests that despite many people’s intuitions to the contrary, the woman has little reason based on the interests of the children she would create to choose to wait. For, whilst many of us tend to believe that it is better to be born without a disability than with one, as disability is, by definition, disadvantageous, person-affecting morality actually gives us little reason based on the interests of the child created to wait to conceive.

If one accepts the TDC2 and holds too that our actions only have moral status when they affect the interests of distinct numerical persons, one can see that regardless of the decision she makes, her child will not be harmed as different children will come to exist depending upon her choice. For, whilst it is true that if she conceives now, her child will be born with congenital syphilis—an undoubtedly harmful condition which may result in cerebral palsy, hearing loss, and musculoskeletal deformities—should she wait to conceive a child free of syphilis, a different child will be born as ‘a different egg and sperm will be involved, resulting in a genetically different individual’ [1]. The child the woman could conceive now could not exist without suffering from congenital syphilis. Being brought into existence suffering from such a condition cannot harm him. For, whilst he might rationally prefer a life without his affliction, the alternative for him is not a life without the effects of congenital syphilis, but no life at all, as syphilis is a condition of his very existence.

Here, however, is where Smajdor takes her position to depart from Parfit’s. For, whilst she agrees that in the case of congenital syphilis, the woman would not harm her child by bringing him into existence with this affliction, non-identity statements are not always so simple.

For although this is not always the case, such statements often come with a qualifier that warns against the creation of lives that are ‘empty of all the things that make life worth living’ [2], ‘dominated by pain and suffering’ [3], ‘intractably miserable’ [4], ‘not worth living’ [5], ‘worse than no life at all’ [6]. This qualifier can be found in the works of many authors who write on the non-identity problem. Smajdor, for example, notes that Robertson suggests that although it is normally the the case that ‘a child’s interests are hardly protected by preventing that child’s existence…, this objection would not hold if the … conditions of his life would be so harmful to him that from his perspective he would prefer not to live’ [15, p. 75].

It is this qualifier with which Smajdor takes issue. For, she holds that its addition entails a commitment to a questionable view: the view that ‘a child born with a worse disease than congenital syphilis *could* have been harmed—if the disease is so terrible as to mean that she does not have a minimally acceptable quality of life’ [1]. In other words, she claims that to add this qualifying phrase entails a commitment to the view that conception can constitute a harm to the child in the latter but not the former case because ‘a threshold has been passed … which was not breached in the case of the child with congenital syphilis’ [1]. Despite the fact that Smajdor fails to truly unpack the threshold account of harm as it is said to apply to non-identity cases, her characterisation of the account is accurate albeit bare. For, those who subscribe to a threshold account of harm in such cases should be seen to hold that some procreative choice made at T_1_ harms a person if and only if it causes into existence a person who falls below some normatively defined threshold of wellbeing, interest satisfaction, etc.

For Smajdor, such accounts are deeply problematic as she is sceptical about the existence of a ‘logical connection between the assertion that some lives are not worth living, and the claim that such people are harmed by being conceived’ [1]. After a rather long section in which she notes the seeming arbitrariness of a threshold account of harm and a number of troubling conclusions associated with this [1], she provides support for her scepticism by asking the reader to consider Kant’s refutation of Anselm’s ontological argument for the existence of God.

In his *Proslogion,* Anselm claims to derive the existence of God from definition: the concept of God as ‘something than which nothing greater can be thought’ [16]. He held that if such a definition is true, God must exist in reality, since if He does not, a greater being can be conceived of: one than which nothing greater can be thought, and which, in fact, exists. Kant however, argued that Anselm committed a grave error in his argument, suggesting that although Anselm treats existence as a property that things may either possess or fail to possess, to say that some entity exists is not to confer existence on it. Instead, it is to say that the concept of the thing is exemplified in the world, just as to say that some entity does not exist is not to state that a thing lacks the property of existence but to say that the concept of that thing is not exemplified in the real world. In other words, Kant argued that a God that exists is identical to a God that does not. For, whether something exists or does not exist does not add to or alter the properties it possesses, it is the mere positing of a thing: ‘a hundred real thalers do not contain the least coin more than a hundred possible thalers’ [17, pp. B626-7] and ‘the real God is not a few degrees more perfect than a conceptual one’ [1].

With this done, Smajdor suggests that we may straightforwardly apply Kant’s argument to the act of creation and the question of whether an entity can be harmed by creation itself. For, she notes, ‘the act of creation can be construed uncontroversially as the act of bringing something into existence,’ and thus, ‘whatever is logically true of existence in general should be true of creation insofar as it is the conferring of existence.… If existence itself cannot entail any other property, then it follows that the mere act of giving existence cannot encompass the act of conferring any property—other than existence—on the entity which is created’ [1]. With this in mind, Smajdor concludes that creating something, or bringing it into existence, cannot be the same as harming it. For, although harm is not a predicate in the usual sense, the fact of having a harmful genetic constitution is. It qualifies an entity in the same way as other predicates. Thus, she suggests that ‘if we cannot make X greater, more perfect, or more valuable by bringing it into existence, neither can we harm X by bringing it into existence however greatly X must suffer’ [1]. She therefore concludes:

There is no specific act that can be construed as harming a future child when the child’s condition is directly linked with the circumstances surrounding its conception. Nor can these questions be a matter of degree, since the logical and metaphysical constraints that prevent us from concluding that a child born with a moderate amount of suffering has been harmed apply equally to all cases of creation. [1]

## Is Smajdor’s characterisation of Parfit’s position fair?

With the major components of Smajdor’s argument explained and laid out above, we now return to the point at which she takes her position on the possibility of harming by the act of creation to differ from Parfit’s. For, we contend that the characterisation she offers of Parfit’s view on the matter of harms to individuals with ‘lives that are less than worth living’ is, at best, uncharitable, and at worst, based on a misunderstanding of the claims made by Parfit on this matter.

The point at which she holds her position to diverge from Parfit’s occurs just after an acceptance of the conclusions of the non-identity problem in the case of the child with congenital syphilis. For, as is noted above, she and Parfit agree that in this case there is little reason for the woman to choose to wait based upon the interests of the child she creates. Her choice is a choice between lives as opposed to a choice of whether to harm or benefit her child. However, whilst this is so, Smajdor argues that Parfit does not discount the possibility that there is a point at which the woman’s child could be harmed by his conception: the point at which this child’s life would be less than worth living, dominated by suffering, or where the quantity and quality of whatever it is that makes life worth living falls below some threshold level, such that the harms he would suffer throughout his life outweigh its pleasures.

Smajdor, however, denies that this could be the case, holding that even if the woman’s choice was between a worthwhile and a less than worthwhile life, no harm would be done to the child created should she choose to bring to birth a child whose life falls below this threshold level. She characterizes Parfit’s view regarding lives that are dominated by suffering as follows: ‘that the child could not have existed without that condition does *not* prevent us from concluding that [he] has been harmed. A threshold has been passed in the latter case, which was not breached in the [former] case’ [1].

She then explores what Parfit might mean by this: asking *why* Parfit should hold that in the case of congenital syphilis the child created cannot be harmed by his mother’s choice, but would be harmed should he suffer more seriously. What, she asks, is it that makes the suffering of the child bear on questions of the morality of the mother’s actions in the latter but not the former case? Why does Parfit seem to divorce the concept of harm from its normal relationship with suffering, turning it into a mercurial entity that flashes into existence only in certain very specific circumstances? [1]

After all, Parfit claims, as do many others who accept the conclusions of the non-identity problem, that non-identity is a logical and metaphysical problem as opposed to one relating to the degree of suffering an individual must endure before he can be deemed harmed, all things considered. The mother is—according to its logic—not morally responsible ‘because there is no causal mechanism by which we can understand him to be harmed’ [1]. Her blamelessness has nothing to do with the fact that her child does not suffer enough or because the harms of his existence fail to outweigh the pleasures his life contains. Her child is not harmed and she may not be criticized morally because he could not have existed absent his condition and she could have done nothing to alleviate his suffering without causing some other child to exist instead of him.

Smajdor thus notes that due to this there is no reason to assume that the conclusions of the non-identity problem should not apply equally in the more serious case. The relevant facts are, she contends, the same in both, regardless of the extent to which the created child suffers once brought into existence. Thus, she holds that Parfit and others are guilty of contradicting themselves when they state that in cases of ‘lives less than worth living’, the child created could be harmed as they are essentially asserting that despite their logical position, ‘existence cannot harm someone … [but] it is, after all, a question of degree’ [1].

Yet, whilst Smajdor’s point is well made—the non-identity problem does seem to apply equally to cases of worthwhile and less than worthwhile lives as the facts relevant to its generation are the same—at no point within *Reasons and Persons* does Parfit suggest that this is not so. He does not claim, despite Smajdor’s assertion, that the child created in the more serious case can be harmed by the act of his creation because his life is less than worth living. He leaves this open to interpretation just as he leaves open to interpretation the question of whether causing some person to exist can be said to cause them some peculiar benefit. He does this in virtue of the fact that he views both a negative and positive answer to the question of whether existence can constitute a predicate as being defensible [10, p. 358].

It should be noted too, in fairness to Smajdor, that because of this, Parfit does not make clear that the conclusions of the non-identity problem may still hold in cases of less than worthwhile lives dependent on the views one holds regarding this. All of his non-identity cases, for example, regard unquestionably worthwhile lives and thus say nothing about the conclusions of the non-identity problem in cases of lives which are less than worth living. Parfit considers, for example, a child whose mother had him too young but whose life, despite its ‘bad start…, [is] predictably worth living’ [10, p. 358]; a choice between conserving our resources for future generations or depleting them such that future generations will live lives of much lower but still acceptable (although barely so) quality [10, pp. 361-4]; and a woman who must choose between waiting to conceive or having a child with a painful but not terrible disability [18]. In other words, although Smajdor asserts that Parfit’s claim is based on a mistake—the formation of an untenable connection between the assertion that some lives are not worth living and the claim that such people are harmed by being conceived—the mistake here seems to be her own. The claim she attributes to Parfit can, on a close reading, be found nowhere in the work she references although, as can be seen in appendix G of *Reasons and Persons*, he is sympathetic to this view.

## Can a child be harmed by his own conception? On comparative and non-comparative accounts of harm in genesis cases

Smajdor seems to suggest that the only plausible accounts of harming are those that are comparative in nature. That is, she seems to recognise as proper only accounts of harming that compare some particular numerical entity’s current state of welfare, happiness, interest satisfaction, etc. to the state that he would have been in had some particular action not occurred. Examples of such accounts are that of the diachronic account, according to which some particular action (or inaction) done at time t_1_ is harmful *iff* it causes some person (p) to be worse off at some later time t_2_ than they were at t_1_, and the subjunctive historical account made famous by Joel Feinberg, which suggests that some particular action (or inaction) done at t_1_ is harmful for p *iff* it causes p to be worse off at t_2_ than he would have been at t_2_ had it not occurred [5].

Such accounts necessarily exclude the possibility that present persons may harm future and merely possible persons by acts of creation in cases of both worthwhile and less than worthwhile lives. This is so because in order to make a harm claim on comparative accounts, one is required to compare the state of p’s interests, welfare, happiness, etc. with the state he would have been in had the act not been performed (subjunctive historical) or was in prior to the performance (diachronic). Thus, as the alternative in non-identity cases is non-existence and non-existence is no state at all, there can be no way to make such a comparison. The writing is, on such accounts, already on the wall as it is nonsensical to discuss a harm threshold when its existence is precluded by the nature of the account in question.

Yet, that this is so does not preclude the possibility that comparative accounts may be compatible with the following argument: although a child who will have a less than worthwhile life may not be harmed by his conception, he may be harmed if his suffering does not end as soon as it begins (whether before or after birth), and this may constitute a good reason not to conceive such a child in the first place. This is not the question addressed by Smajdor in her article, and as such, it has been understandably glossed over. However, it does seem important. For, it means that on comparative accounts, although we may not say that a child can be harmed by his conception even if his life is one that is less than worth living, we can provide good person-affecting reasons to support a moral requirement to prevent the suffering of a child whose life will be dominated by suffering.

For, if there exists a moral duty not only to refrain from causing persons to suffer but also to actively seek to ameliorate suffering where it occurs, we may find that in cases of lives dominated by suffering, there is a moral duty to end the lives of such individuals as soon as they come to suffer. We need not claim that the child created is harmed by his own conception or that those responsible for his conception are to blame for his poor prospects. He is not and they are not, as nothing could have been done to avoid this unfortunate stacking of the deck. Yet, whilst the prospective child may not be harmed by his conception, we may hold that those responsible for his conception are morally blameworthy for failing to stop his experience of such severe and uncompensated suffering in the face of full knowledge of his devastatingly poor life prospects.

Consideration of the nature of different accounts of harm seems to uncover another major problem inherent in Smajdor’s article. This can be found in the fact that it is poor philosophical practice to attempt to criticise one account by merely showing that it is different from another. That such is the case is already transparent. The utilitarian may not fairly criticise the Kantian by stating only that he is not a utilitarian. He must instead uncover some fatal flaw in the theory of his foe or appeal to good reasons suggesting his account is more plausible. With this in mind, we can note that those who subscribe to comparative accounts of harm cannot refer to the inadequacy of non-comparative theories by merely noting that they are not the same as their own. The threshold account is a non-comparative theory. As such, what is needed on Smajdor’s part is an explanation of *why* the particular account of harm she seeks to criticise should be seen to be lacking in some sense, and this can only be done via explication *or* the provision of an explanation of *why* one’s own account overcomes such shortcomings by demonstrating both its superiority and its avoidance of other equally troubling conclusions.

Comparative accounts are, however, not the only plausible accounts of harm to which one may appeal. To be sure, they are intuitive, since generally, when we think of harm, we think of things going worse for someone than they otherwise might have. Yet, despite this, such accounts are not a panacea. They face a number of problems that are not limited only to the fact that they fail to account for our intuitions in non-identity cases. Thus, although we might be willing, as Smajdor is, to bite the bullet in such cases and accept the counterintuitive conclusions comparative accounts engender in such cases, these are not the only counterintuitive conclusions such accounts are often argued as entailing. Comparative accounts have been said, for example, to fail to account for circumstances in which we wish to say that an individual has been both significantly harmed and greatly benefited by an act which causes an on-balance benefit, such as in Seana Shiffrin’s example of the generous but dangerous millionaire who drops large blocks of gold bullion from the sky as gifts to citizens of a neighbouring town where the million dollar manna ends up falling on and injuring one of the recipients [19]. They are charged with multiplying harms excessively due to the fact that, on such accounts, harms are grounded in comparisons, leading to confusing and questionable determinations. For when one is harmed, one does not undergo a separate harm relative to each earlier moment in one’s life at which one fared better [20], as would be the case on the diachronic comparative account, and neither does one undergo a number of different harms in cases where one is shot just because one would have been better off had one’s assailant not pulled the trigger, had the gun not gone off, or had the bullet missed one’s body, as would be the case on the subjunctive historical account [20]. Comparative accounts, it has been said, fail to account for the harm in beneficial cases of self-harming, as in the case of the ‘Blighty’ wound [7, p. 92],^2^ and for pre-emptive harms [20]. Indeed, whilst this is not the place for a lengthy discussion of these shortcomings, they are numerous and have been widely discussed within the literature.

One account of harm often presented as a credible alternative to comparative accounts, and which seems to overcome a great number of the problems often associated with them, has been championed by one of the authors of this commentary, Harris. It is set out in great detail in his book *Wonderwoman and Superman* and is often termed the ‘harmed state account’. On this account, and on other similar accounts of harm such as that proposed by Shiffrin [19], the notions of harming and wronging set out in comparative accounts of harm seem to be turned upside down, and a particular numerical entity can be said to be harmed when it is simply the case that he has been put into a condition that is harmful. As Harris explains:

A condition that is harmful … is one in which the individual is disabled or suffering in some way or in which his interests or rights are frustrated. The disability or suffering may be slight, just as harms are trivial…. I would want to claim that a harmed condition obtains wherever someone is in a disabling or hurtful condition, even [if] that condition is only marginally disabling and even [if] it is not possible for that particular individual to avoid the condition in question. [7] p. 88]

On this account of harm, therefore:

To suffer harm is to come to be in—or perhaps better, is simply to be in—a certain sort of non-comparatively bad state. It is to come to be in … a state in which one fares, not worse than one fared, or would have fared, in some alternative state of affairs, but simply badly. The seriousness of a given harm, according to this way of thinking, is proportionate to the (non-comparative) badness of this state. [20]

Such accounts therefore leave open the possibility of all sorts of harms befalling future and present individuals even if they cannot be said to have been made worse off. Quite often, such individuals will, of course, be made worse off, but this is not a central question. As such, the possibility is left open that individuals can, and quite often *will*, be deemed harmed in non-identity cases, because although they would never have come into existence had the act not been done, in order to determine harm, one needs only to point to the fact that the individual suffers.

Returning to the congenital syphilis case, then, we note that should the woman choose to conceive now, she will conceive a child who suffers from the effects of congenital syphilis, and thus, her child will be born harmed by her action. Despite the fact that had she made a different choice he would not exist, she is still to be held responsible for the harm that has occurred as ‘where B is in a condition that is harmed and A and/or C is responsible for B’s being in that condition, then A and/or C have harmed B’ [7, p. 89]. Non-identity does, however, still represent a problem on such accounts if we are unwilling to appeal to impersonal harms and wrongs^3^ in cases such as the above, resulting in harmed but still worthwhile lives, but it occurs for the reason that no wrong can be determined. It remains a problem:

Not because the life in question has not been impaired, not because the individuals are not suffering, not because they have not been harmed: it has, they are, and they have: rather because it is not possible to regard them as having been wronged. You might harm someone in order to benefit them, but if so, you do not wrong them unless you violate their will in order to do so or breach some other obligation to them. The mother giving a life with some measure of disability to a child who will find such a life worth having does not wrong her child. She is like the doctor giving a drug [that] has damaging side effects but side effects [that] are worth enduring for the sake of staying alive. [7, p. 95]

In the congenital syphilis case, the child *should* be seen as harmed by his mother’s act, but the mother is saved from blameworthiness *because* her child has a life that is worth living. He will not be wronged by her because, ‘like those with Blighty wounds or those who have to endure the harmful side-effects of beneficial drugs, [he] has received a net benefit from what has happened to [him] and none of [his] rights have been violated’ [7, pp. 95-6]. It would be irrational for him to condemn his mother for her choice as he would not have existed without his condition and his existence is, although not perfectly so, pleasurable.

Yet, returning to the more serious case of the choice to bring to birth a child whose life will be less than worth living, we note that non-comparative accounts can state that to make such a choice, in full knowledge of this fact, constitutes both a harm and a wrong.^4^ In other words, non-comparative accounts are, unlike comparative accounts, compatible with a subscription to a harm threshold. This, of course, requires an answer to the question of what it actually means for an individual to be in a worse off condition than non-existence, and to make a determination of where this threshold lies. However, despite Smajdor’s protestations, an answer can be easily found. For, although non-existence is not a state of being and thus cannot be said to be preferable *for* an individual, it can be in an individual’s interests to end her life or to have her life ended for her. If it were not, we would not view it to be ‘better’ to painlessly end the life of a suffering animal than to allow it to continue existing in a state that is both terrible and terrifying for it, and neither would we seriously consider questions relating to rational suicide or voluntary euthanasia. As Parfit notes:

A certain kind of life may be judged to be either good or bad—either worth living or not worth living. If a certain kind of life is good, it is better than nothing. If it is bad, it is worse than nothing…. Consider someone dying painfully who has already made his farewells. This person may decide that lingering on would be worse than dying. To make this judgement, he need not *compare* what it would be like to linger on with *what it would be like to have died* … he might consider what lay before him, and decide whether he did or did not want to undergo it. [10, p. 487]

With this in mind, it is held that for existing individuals, the question of what constitutes a less than worthwhile life can only be answered subjectively, for in such cases we ask it in regards to ‘individuals who can have a view about the desirability of their own existence’. In this sense, ‘a condition is worse than non-existence if and only if the subject would rather not exist than exist in such a condition’ [7, p. 93]. Where the threshold lies for conception to be considered wrongful is trickier to determine, however. For in such cases, we cannot ask the child to be created whether he would consider his life worth living, and nor may we appeal to the conditions under which we would consider our own lives to be less than worth living. Instead, we must attempt to determine, in light of the information available to us, whether the child to be created is likely to consider his life to be worth having, and this is to be done by assessing ‘whether or not such a life has a favourable balance of satisfactions over miseries’ [7, p. 93].

If we turn, then, to Smajdor’s concerns regarding whether existence can constitute a predicate, we can note that it is not at all clear how such considerations bear on the question of harmful conceptions and wrongful lives on threshold accounts. For on such accounts, whether we do or do not choose to bring to birth a child with a harmful genetic constitution has little bearing on the properties with which he would enter the world. To be sure, non-existent things lack all properties, as existence is a precursor for having properties, and thus, to state that a child can be harmed or benefited by not being brought into existence is nonsensical; however, existent things can, on non-comparative accounts of harm, possess as a necessary property the property of harm itself. For example, we can note that in the congenital syphilis case, whether or not the mother decides to birth the child/children she could have before the cure, it will be deemed that his/their necessary properties are harmful as such properties result in suffering. The harm is inherent in that child’s necessary properties, and thus, the concept of the child with congenital syphilis already contains harm, which is not altered in the least bit by our choice of whether or not to bring him to birth or by whether we view existence to constitute a predicate.

Indeed, the corollary on such accounts seems to confirm this: for, non-comparative accounts such as Harris’s also hold that if an effective treatment were discovered after the child’s birth it is the harm that the child experiences which provides the motive for administering the treatment. If the child were not in a harmed condition, if the condition is not harmful, then why attempt to treat the congenital syphilis or seek to discover a cure?

The same, incidentally, goes for understanding the moral imperatives engaged by the possibility of human enhancement, the possibility of improving on normal species functioning or species typical functioning. The moral motive for human enhancement is generated by the possibility of ameliorating the human condition, of seeing the harmfulness of things as they are, the imperfections of human nature. If there is such a thing as human nature, this ‘fact’ does not prevent us from seeing its limitations and harmful effects, even when we have no different extant states of being with which to compare. The possibility of enhancing evolution, of improving on human nature, seems, on such accounts, to create a new conception of what it is to be in a harmed condition, not relative to existing alternative states, but to possible enhancements [21, pp. 86-109].

## Conclusion

We have considered a number of arguments forwarded by Anna Smajdor in her article How Useful is the Concept of the “Harm Threshold” in Reproductive Ethics and Law?’ where she sought to uncover a number of problems inherent in the concept of the harm threshold by appealing to Kantian arguments concerning the question of whether existence can constitute a predicate. After exploring and explaining Smajdor’s arguments, we acknowledged that the question of whether existence can constitute a predicate is relevant for questions of the ethics of reproductive decision making on comparative accounts of harm, and thus, that the concept of the harm threshold lies on shaky ground on such accounts. However, while such is the case, we have also shown that Smajdor’s characterisation of Parfit’s position on the possibility of threshold harms in non-identity cases rests on a mistake and that the arguments to which Smajdor appeals do not seem to apply on non-comparative accounts of harm, which, incidentally, tend to be the accounts of harm to which those who actually appeal to the concept of a harm threshold subscribe.

Smajdor, therefore, fails to provide compelling reasons for those who subscribe to non-comparative accounts to abandon their claim that in cases of lives less than worth living, persons can be both harmed and wronged by being brought into existence. With this in mind, we suggest that should Smajdor wish to continue her work in this area, she must try to engage more fully with non-comparative accounts of harm and provide arguments giving those who subscribe to such accounts reasons to abandon their view. For, if she believes, as her article seems to suggest, that we may only appeal to comparative harms when making ethical and legal decisions, such a position cannot be defended along the lines she proposes.

## Footnotes

1 Provided they accept some version of the claim that our existence is dependent on our being conceived from the gametes from which we are actually conceived.

2 The term ‘Blighty wound’ is often used to refer to a wound (often self inflicted) sustained by a soldier during wartime that is serious enough to get him sent home from the battlefield but not so serious as to kill or reduce his quality of life in any significant way, thus, benefiting him overall.

3 Broadly defined as those resulting from the bringing of avoidable suffering into the world.

4 Note that in cases where the decision to bring to birth lives that are highly likely to be less than worth living for reasons other than the child’s own limitations, such as for reasons of social justice, there may be other proper subjects of blame than the mother. Consider, for example, the choice to bring to birth a black child in a highly racist society or a case in which an individual would have a life that is worth living but for the denial of the resources that should, in accordance with one’s particular theory of justice, be provided to him.

## References

[1] Smajdor, Anna. 2014. How useful is the concept of the ‘harm threshold’ in reproductive ethics and law? *Theoretical Medicine and Bioethics* 35(5) [Epub ahead of print]. 10.1007/s11017-014-9302-8 PMC4174351 25106477

[2] Steinbock Bonnie, Roberts Melinda A, Wasserman David T (2009). Wrongful life and procreative decisions. Harming future persons: Ethics, genetics and the nonidentity problem.

[3] Bennett Rebecca (2009). The fallacy of the principle of procreative beneficence. Bioethics.

[4] Rakowski Eric (2002). Who should pay for bad genes?. California Law Review.

[5] Feinberg Joel (1986). Wrongful life and the counterfactual element in harming. Social Philosophy and Policy.

[6] Brock Dan W (1995). The non-identity problem and genetic harms: The case of wrongful handicaps. Bioethics.

[7] Harris John (1990). Wonderwoman and superman.

[8] Williams Nicola J (2013). Possible persons and the problem of prenatal harm. The Journal of Ethics.

[9] Kavka Gregory, S (1982). The paradox of future individuals. Philosophy & Public Affairs.

[10] Parfit Derek (1984). Reasons and persons.

[11] Belshaw Christopher (2000). Identity and disability. Journal of Applied Philosophy.

[12] Wrigley Anthony (2012). Harm to future persons: Non-identity problems and counterpart solutions. Ethical Theory and Moral Practice.

[13] Heyd David, Roberts Melinda A, Wasserman David T (2009). The intractability of the nonidentity problem. Harming future persons: Ethics, genetics and the nonidentity problem.

[14] Thompson Janna (2000). The apology paradox. Philosophical Quarterly.

[15] Robertson John A (1994). Children of choice: Freedom and the new reproductive technologies.

[16] Anselm. 2000. Proslogion. In *The complete philosophical and theological treatises of Anselm of Canterbury*, trans. J. Hopkins and H. Richardon. Minneapolis: Arthur J. Banning Press.

[17] Kant Immanuel (1929). Critique of pure reason.

[18] Parfit Derek, Singer Peter, Kuhse Helga (2006). Rights, interests and possible people. Bioethics: An anthology.

[19] Shiffrin Seana Valentine (1999). Wrongful life, procreative responsibility, and the significance of harm. Legal Theory.

[20] Hanser Matthew (2008). The metaphysics of harm. Philosophy and Phenomenological Research.

[21] Harris John (2007). Enhancing evolution.

